# Site suitability assessment for the development of wind power plant in Wolaita area, Southern Ethiopia: an AHP-GIS model

**DOI:** 10.1038/s41598-023-47149-x

**Published:** 2023-11-13

**Authors:** Natei Ermias Benti, Yewubdar Berhanu Alemu, Mathewos Muke Balta, Solomon Gunta, Mesfin Diro Chaka, Addisu Gezahegn Semie, Yedilfana Setarge Mekonnen, Hamere Yohannes

**Affiliations:** 1 Computational Data Science Program, College of Natural and Computational Sciences, Addis Ababa University, P. O. Box 1176, Addis Ababa, Ethiopia; 2 Space Science and Geospatial Institute, Addis Ababa, Ethiopia; 3 Department of Environmental Science, College of Natural and Computational Sciences, Wolaita Sodo University, P.O. Box 138, Wolaita Sodo, Ethiopia; 4 Department of Physics, College of Natural and Computational Sciences, Wolaita Sodo University, P.O. Box 138, Wolaita Sodo, Ethiopia; 5 Center for Environmental Science, College of Natural and Computational Sciences, Addis Ababa University, P. O. Box 1176, Addis Ababa, Ethiopia; 6 School of Civil and Environmental Engineering, Addis Ababa Institute of Technology, Addis Ababa University, Addis Ababa, Ethiopia

**Keywords:** Energy infrastructure, Renewable energy

## Abstract

The primary driver of economic growth is energy, predominantly derived from fossil fuels, the demand for which has experienced a significant increase since the advent of the Industrial Revolution. The emissions of hazardous gases resulting from the utilization of these fuels have been well acknowledged, therefore exerting a notable impact on the environment. In the context of Ethiopia, it is observed that despite the presence of ample renewable resources, the accessibility to power continues to be constrained. In order to effectively tackle this issue, it is imperative to redirect attention towards the utilization of renewable sources, such as wind energy, as a means of enhancing the existing power grid infrastructure. The present study used geospatial tools to evaluate the appropriateness of the Wolayita region for the establishment of a wind power facility. The process of site selection is guided by multiple factors, and a multi-criteria approach is facilitated through the utilization of Geographic Information System (GIS). The evaluation of seven characteristics was conducted utilizing the Analytical Hierarchy Process (AHP) methodology, which involved pairwise comparisons and weighted scoring. The process of suitability mapping involves the classification of locations into four distinct categories, which range from the most suitable to the least suitable. The findings demonstrate that the area of 0.628% (28.00 km^2^) is deemed the most suitable, while 54.61% (2433.96 km^2^) is considered somewhat acceptable. Additionally, 0.85% (37.85 km^2^) is identified as the least suitable, leaving a remaining 43.91% (1060.00 km^2^) that is deemed unsuitable. The central, northwestern, and southern regions are identified as optimal geographic areas. The results of this study facilitate the process of investing in renewable energy, thereby assisting Ethiopian authorities and organizations in promoting sustainable development. This report serves as a crucial reference point for the wind energy industry.

## Introduction

In today’s era of rapid population growth and industrial expansion, global energy demands have undergone unprecedented changes^1^. Since the Industrial Revolution, the widespread consumption of conventional fossil fuels has triggered the release of various greenhouse gases (such as CO_2_, CH_4_, etc.), resulting in an unprecedented increase in average global temperatures and environmental concerns^2–4^. These traditional fuels have inflicted detrimental impacts on the environment, economy, and human health, including deforestation, environmental pollution, and the depletion of soil nutrients due to increased reliance on agricultural waste and animal dung. The combustion of dung and wood as cooking fuels has escalated the risk of acute respiratory infections. As a response, the prioritization of environmentally friendly energy sources has become imperative^5^.

Renewable energy sources, often referred to as eco-friendly alternatives, have emerged as primary replacements for traditional energy resources, presenting the potential to meet escalating energy demands^4,6–8^. These sources, encompassing wind, solar, hydro, biomass, and geothermal energy, are abundant, clean, and free, contrasting finite fossil fuel reserves^9–12^. Recognized for their environmental suitability, renewable energy outperforms conventional systems by mitigating air pollution inherent in fossil fuel combustion. Among these, wind and solar energy have reached technological and economic maturity, with wind energy standing out due to its cost-effectiveness, efficient multi-megawatt turbines, and accessibility^12^.

Globally, wind energy installations have thrived across more than 95 countries, resulting in an estimated global wind capacity of 539.58 GW by the close of 2017^13^. Denmark boasts the highest wind power penetration with a 40% market share, followed by Uruguay, Ireland, Portugal, Cyprus, and Spain, all exceeding 20% penetration. Germany stands at 16%, while Canada, China, and the United States hold 6%, 5.5%, and 4% respectively^13^. In Africa, South Africa, Egypt, and Morocco lead in wind energy production, possessing capacities of 2094 MW, 810 MW, and 787 MW respectively^13^. Despite Ethiopia's abundant renewable resources, including wind, solar, hydropower, geothermal, and biomass, their utilization remains limited. The predominant energy source is hydro, accounting for 86% of the country's electricity generation^14,15^. In an effort to diversify its energy portfolio and reduce dependency on hydropower, Ethiopia is expanding wind energy initiatives. This is due to the complementary nature of wind and hydro energy. Wind energy projects are planned, with short, medium, and long-term targets of 970 MW, 1750 MW, and 4000 MW respectively. However, the actual installed capacity is 324 MW, sourced from three wind farms: Adama I (51 MW), Adama II (153 MW), and Ashegoda (120 MW)^14,16^.

Site selection for wind energy projects demands meticulous consideration of diverse variables, integrating environmental, economic, and land use factors. Optimal sites must be chosen based on a spectrum of economic, ecological, and physical criteria, transcending mere wind intensity^17^. Multi-criteria decision-making (MCDM) methods, encompassing techniques such as the weighted sum method (WSM), Analytical Hierarchy Process (AHP), weighted linear combination (WLC), and more, are employed for wind farm location assessments^18,19^. Among these, AHP stands out for its simplicity, ease of use, and capability to evaluate decision consistency^20^. AHP allows for the incorporation of both qualitative and quantitative criteria, empowering decision-makers to assign relative weights based on their expertise^21,22^. Previous studies have successfully combined AHP with Geographic Information System (GIS) to select wind energy sites based on various factors^23–25^.

Despite several studies exploring wind energy potential, the process of site selection in Ethiopia's Wolaita area remains unexplored. This study presents the first comprehensive analysis employing AHP and GIS to identify potential wind farm sites in the region. We complement this investigation with extensive literature reviews and expert input, fortifying the AHP estimation. Given its nationwide scope, this research is anticipated to provide valuable insights for the broader application of wind energy on a national scale. This alignment with sustainable development goals (SDGs) ensures that our findings contribute to sustainable and well-informed energy planning and implementation.

## Materials and methods

### Description of the study area

**Figure 1:**
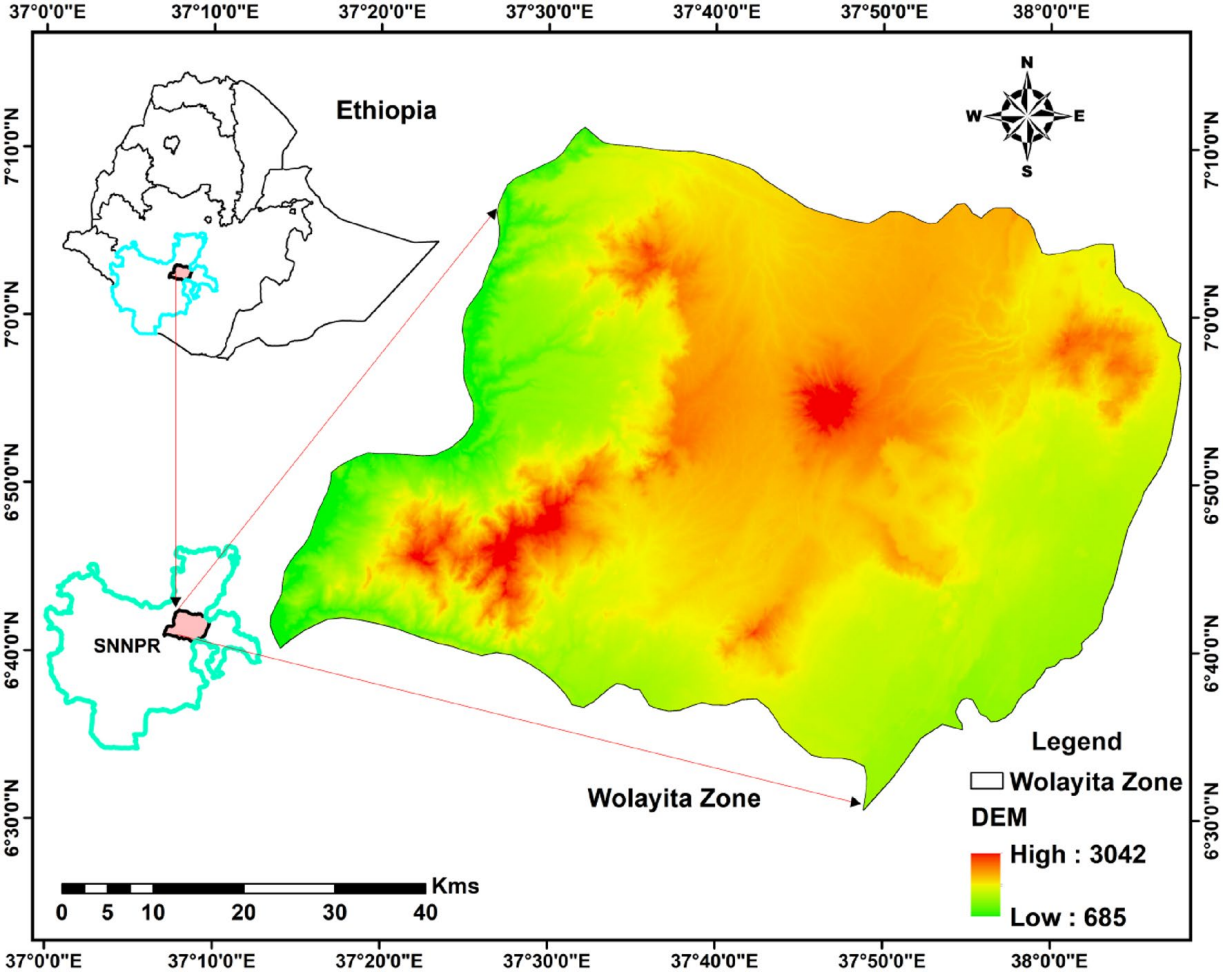
Location map of the study area.

The Wolayita Zone is located in Southern Ethiopia between the coordinates 6.4–7.1° N and 37.4–38.2° E, with the lowest elevation of 1500 m above sea level and the highest elevation of over 3000 m at Damota Mountain. The Zone is found about 300 km south of Addis Ababa (Fig. 1). It is bordered on the south by Gamo Gofa, on the west by the Omo River, which divides it from Dawro, on the north by Kembata Tembaro and Hadiya, on the east by the Bilate River, which separates it from Sidama, and on the south-east by the Lake Abaya, which separates it from Oromia region^26^. The area experiences a subtropical highland climate with year-round average temperatures of 24°–30° C during the day and 16°–20° C at night. The major rainy season is from June to September, with an annual average rainfall of 1350 mm^26^.

### Data types and sources

In this study, our attempt entails the integration of AHP-GIS spatial analysis to assess the suitability of wind farm locations within Ethiopia’s Wolayita region. The geo-information data were gathered from various organizations (Table 1). This study uses seven evaluation criteria to identify the best locations for wind farms and the worst locations. The suitability map was produced using ArcGIS 10.5^27^.

**Table 1:** Data types and sources used in this study.

Data	Data type	Purpose	Data source
Boundary	Shape file/polygon	To evaluate a focus area	EthioGIS from https://www.wlrc-eth.org
Wind Speed	Statistical data	To determine the potential for wind energy	Global wind atlas
Settlements	Shape file/polygon	To lessen the visual effect and noise pollution	Derived from the LULC map
Power lines	Shape file/polyline	To decreases in the cost of energy transfer to the grid	Ethiopian Electric Power (EEP)
Road Network	Shape file/polyline	To place the wind farm close to the road	EthioGIS, https://www.wlrc-eth.org
Rivers	Shape file/polygon	To protect environmentally vulnerable areas	EthioGIS, https://www.wlrc-eth.org
Slope	Raster	To determine whether a wind power construction is feasible	United States Geological Survey (USGS), https://earthexplorer.usgs.gov/srtm/
LULC	Raster Image	To reduce the environmental impact of the development	European Space Agency (ESA), https://data.apps.fao.org/catalog/dataset/378e9df5-3814-4a80-bb47-e25df0543634

### Parameters for assessing land suitability

To conduct a geographical analysis and pinpoint optimal sites for the Wolayita wind farm, a set of seven assessment criteria was formulated and input into GIS environment. These criteria encompassed wind speed, proximity to settlements, spacing between power grids, distance from transmission systems, river proximity, slope, and land use/land cover. These variables were chosen because they directly impact the feasibility and success of wind farm projects. All the considered input parameters were prepared following scientific producers.

#### Wind speed

Wind speeds play a pivotal role in determining the optimal locations for establishing a wind power plant. This is due to the fundamental relationship between wind speed and the efficiency of wind turbines. Wind turbines initiate their operation when wind speeds reach around 3 m/s and automatically shut down when wind speeds exceed approximately 25 m/s. For efficient energy generation, wind speeds of at least 3.5 m/s are considered favorable^28^.

In the context of site selection, ensuring a minimum wind speed of 3.5 m/s is a foundational step. This criterion is employed to filter out areas where wind speeds are expected to fall below the threshold of efficient energy generation. By excluding such locations from consideration, the study can focus on sites with more promising wind resources that align with the operational efficiency of wind turbines. In essence, this initial filtering process aims to identify areas where the wind resource is potent enough to support productive and sustainable wind power generation.

#### Distance to settlement

The proximity of wind power plants to settlements can impose adverse effects on the local population. Concerns include issues such as noise pollution from wind turbines, the discomfort caused by shadow flickers affecting residents, diminished wind speeds, and the potential interference with future residential expansion. Thus, it becomes imperative to maintain an appropriate distance from human settlements. Notably, an optimal distance of up to 7.5 km between wind power plants and residential areas is recommended to mitigate environmental repercussions^29^.

#### Distance from power lines

The primary goal of the wind farm is to generate electricity, which is subsequently channeled into the main grid via transmission lines. Consequently, in selecting an optimal wind farm location, the proximity between potential sites and existing transmission lines, as well as the broader power infrastructure, holds paramount significance. It is imperative to establish a minimum separation distance to mitigate any potential adverse impacts of power transmission on public health^30^. This study has ascertained that a minimal distance of 1.67 km should be maintained between power transmission lines and the designated wind farm sites^31^. In determining this minimum distance, our approach is founded on a comprehensive synthesis of safety regulations, industry standards, and insights from existing literature. This distance is a well-informed choice that prioritizes safety, aligns with established norms, and takes into consideration practical feasibility. Expert consultation in the field of wind energy development further validates this decision.

#### Distance from rivers

Rivers, lakes, and wetlands are examples of water bodies that have been deemed unsuitable for use as wind farm sites due to the ecological services they provide^32^. Water bodies have significant ecological and economic value. They frequently serve as habitats for a variety of very rich and diverse species of flora and fauna. The wind farms' distance from the riverbed will increase the safety of the facilities because the river routes are dynamic and constantly changing, and there is also a risk of flooding^25^. Within 300 m of water bodies, renewable energy projects should not be constructed^25^. A 0.6 km buffer is built around the water bodies in this study. The buffered water course places were subsequently left out of the study area.

#### Distance from transportation lines

The location of wind farms should ideally be as near as possible to main roads in order to minimize multiple discomforts, such as the negative impact on road mobility due to loud noises and changes in the visual scene due to the rotation of the wind turbines caused by wind turbine operation^28^. Furthermore, wind farms should be located as close as possible to main roads to reduce transportation costs and make access easier for various employees. Therefore, 1.67 km should be the minimum distance between the wind farm projects and the main roads^33^.

#### Slope

The slope is a crucial technical factor that must be taken into consideration when selecting wind farms. Because these locations are difficult to access due to steep slopes, maintenance and equipment installation costs increase. The slope raster was extracted from 30 m DEM downloaded from the USGS website (Table 1). For wind farm establishment, flat and low-slope areas are frequently advised to avoid the hurdles of wind farm construction. On the other hand, regions with a slope of more than 10% are excluded from the final suitability map^30^.

#### Land use/land cover (LULC)

One of the key considerations for energy investments is land use. In areas where wind turbines barely impact current land use, wind energy should be installed. Land use has an impact on the choice of a wind farm because there are some locations where wind farms cannot be built even though there is sufficient wind speed, like in a forest, wetland, aviation zone, archaeological site, etc. Therefore, it can be generally said that the most suitable types of land are agricultural land, grassland, barren land, and shrub land, while forest land is considered to be less suitable^23,34,35^. For this study, Land cover data with a 10 m resolution was acquired from the European Space Agency (ESA) and resampled to 30 m to match with the resolution of other inputs. This study did not include wetlands, water sources, or settlements because it would not be appropriate to construct wind farms there^23^.

### Multi-criteria decision-making

The Analytic Hierarchy Process (AHP) has garnered considerable scholarly attention, primarily owing to its robust mathematical underpinnings and its extensive application across a diverse spectrum of disciplines^36^. Esteemed for its adeptness in addressing multifaceted challenges of multi-criteria decision-making (MCDM), the AHP has found a ubiquitous role in the repertoire of researchers hailing from various fields and contexts. When confronted with intricate MCDM dilemmas, the AHP empowers decision-makers by offering a systematic mechanism to apportion weights to influential factors, thus guiding them toward optimal solutions. Orchestrated around a hierarchical model that encompasses objectives, criteria, sub-criteria, and potential alternatives, the AHP method provides a structured avenue for resolving intricate problems^37^. After delineating the problem's architecture, the hierarchy is methodically established, thereby paving the way for a pairwise comparison matrix. This matrix, delineated in accordance with a calibrated preference scale, provides the means to compare criteria across disparate hierarchy levels^38^.

The AHP operates on the principle of pairwise comparison within a measurement framework. It employs an absolute judgment scale, quantifying the degree of dominance of one element over another concerning a specific attribute^39^. The derived priority scales are formulated by multiplying the priority of parent nodes with their corresponding criteria, summing these values across nodes. AHP leverages a reciprocal matrix and an absolute judgment scale to discern the extent of one criterion's dominance over another during pairwise comparisons. The process encompasses two key steps: firstly, establishing the relative importance of each criterion, and secondly, determining the relative weight.

Nonetheless, the AHP method entails a potential for introducing inconsistencies during pairwise comparisons. Thus, an assessment of the consistency underlying these comparisons becomes imperative. To address this,^37^ introduced the consistency ratio (CR) as a metric to gauge the harmony among pairwise comparison-based evaluations^21,37^. CR is computed for the pairwise comparison matrix, and its consistency is affirmed (CR = 0.1) when the total contradictions among comparisons fall below a predefined threshold^40^. If not, a reevaluation of the decision-making process becomes essential. Equation (1) can be used to determine the CR.

1 $$ CR = \frac{CI}{{RI}} ;\;\;CI = \frac{{\lambda_{m} - n}}{n - 1} $$

where λ_m_ is the primary eigenvalue of the comparison matrix, CI is the consistency index, and RI is the random index, which depends on the size of the matrix (n)^37^.

Saaty stipulates an upper limit of “0.10” for this ratio^37^. A CR below 0.10 signifies adequate consistency, warranting the progression of evaluation. Conversely, a CR exceeding 0.10 denotes inconsistencies in judgments, prompting a higher-quality review of decisions. The examination of judgments can aid in reducing CR^40^.

#### Criteria standardization and weight assigning

The acquired input data have different unit and measurement; therefore it is necessary that factors are standardized before combination. In this study, the standardization of factors was undertaken based on literature. Thus, we transform the factors into the same scale based on specified threshold values using reclassification that standardizes the inputs using reclassify spatial analysis tool in ArcGIS environment. The weights of each factor then assessed using MCE (Multi-criteria evaluation) in AHP tool. The factor weight is important to determine the preference of each criterion relative to the other factor effects on the energy production rate.

#### Weighted overlay analysis

The weighted overlay analysis is important to aggregate all the factors/criteria under investigation. All reclassified factors were aggregated and weighted using the function of overlay analysis in in ArcGIS environment, this enable us to perform the overlay analysis and produce the final suitability map.

**Figure 2:**
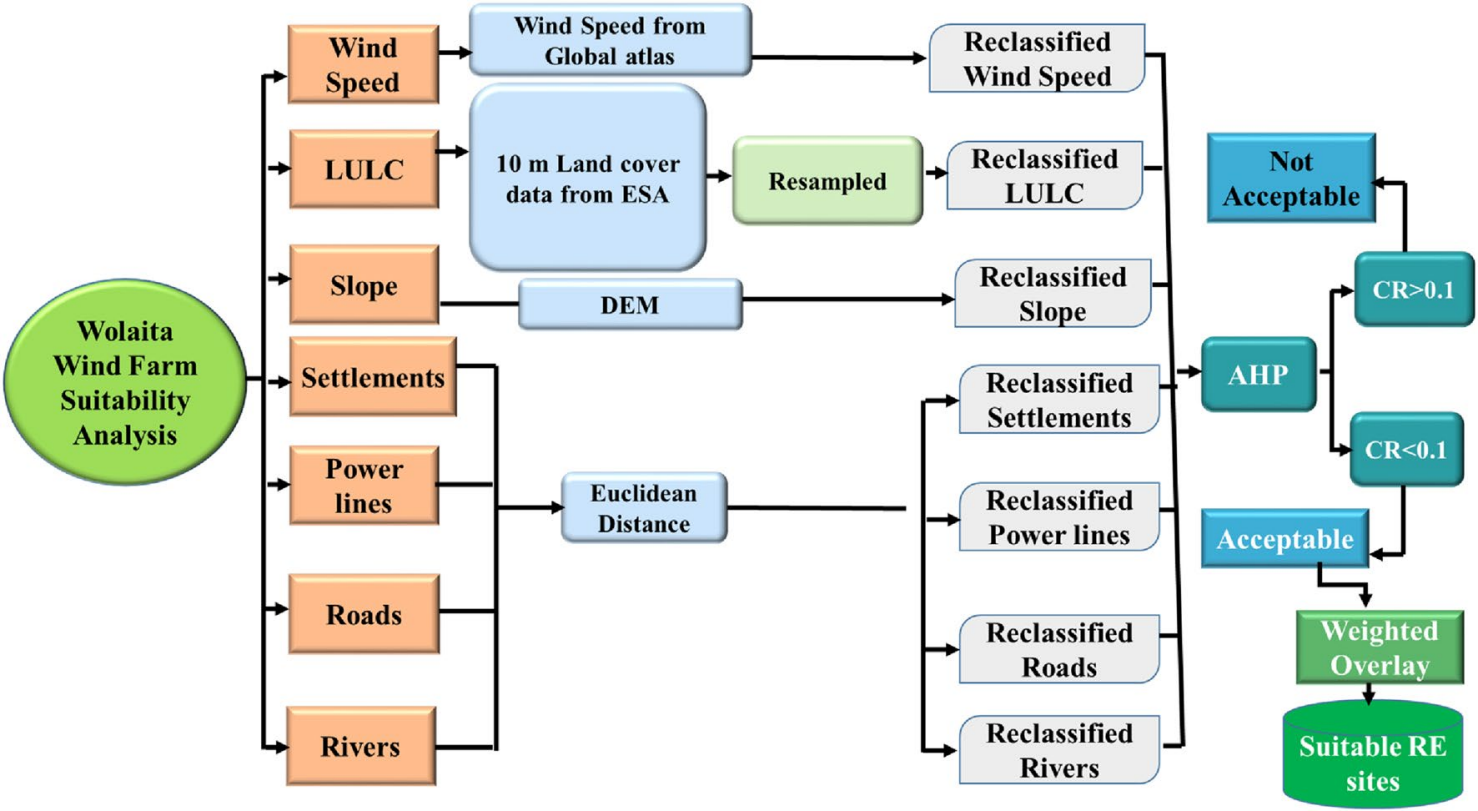
Methodological framework of the study.

The methodological framework for wind farm siting assessment used in this study is broken down into a number of steps, which are described and depicted in Fig. 2.

## Results and discussion

### Suitability of environmental factors

Land suitability mapping is crucial for wind farm planning. This section introduces the criteria and factors essential for evaluating suitable areas. Factors like wind speed, distance to settlements, power lines, rivers, transportation, slope, and land use/land cover are pivotal in making informed decisions for efficient wind energy generation.

#### Wind speed

Wind speed holds significant importance in the process of selecting an optimal location for establishing new wind farms. The energy output of wind turbines exhibits a positive correlation with increasing wind speeds, up to the point of reaching the designated nominal wind speed. This speed level is pivotal for maximizing power generation. Consequently, regions characterized by higher wind speeds are notably preferable compared to those with lower wind speeds.

In the specific context of assessing the suitability of the Wolaita zone for the deployment of wind farms, the evaluation criteria primarily revolved around wind speed. Through an analysis of frequency values and statistical data, land classifications were segregated into four distinct categories: “Most Suitable,” “Moderately Suitable,” “Least Suitable,” and “Not Suitable” (Table 2).

**Table 2:** Suitability score and areas coverage for the selected parameters.

Evaluation factor	Suitability class	Score	Range	Area (km^2^)	Area coverage (%)
Wind speed (m/s)	Most suitable	1	> 3.5	208.99	4.69
	Moderately suitable	2	1.75 to 3.5	2694.98	60.47
	Least suitable	3	0.88 to 1.75	1294.89	29.05
	Not suitable	4	< 0.875	257.87	5.79
	Most suitable	1	> 7.5	1773.98	39.80
Distance to settlement (km)	Moderately suitable	2	5 to 7.5	1049.50	23.55
	Least suitable	3	2.5 to 5	966.79	21.69
	Not suitable	4	< 2.5	666.47	14.95
	Most suitable	1	< 1.67	807.38	18.12
Distance to power lines (km)	Moderately suitable	2	1.67 to 3.34	696.28	15.62
	Least suitable	3	3.34 to 5	599.47	13.45
	Not suitable	4	> 5	2353.61	52.81
	Most suitable	1	> 0.6	3815.96	85.62
Distance to rivers (km)	Moderately suitable	2	0.4 to 0.6	232.51	5.22
	Least suitable	3	0.2 to 0.4	238.98	5.36
	Not suitable	4	< 0.2	169.29	3.80
	Most suitable	1	< 1.67	922.78	20.71
Distance to transportation network (km)	Moderately suitable	2	1.67 to 3.34	757.17	16.99
	Least suitable	3	3.34 to 5	645.63	14.49
	Not suitable	4	> 5	2131.15	47.82
	Most suitable	1	< 10	3373.76	75.70
Slope (^o^)	Moderately suitable	2	10 to 20	741.24	16.63
	Least suitable	3	20 to 30	276.74	6.21
	Not suitable	4	> 30	65.00	1.46
	Most suitable	1	Open Areas & Bare ground	963.39	21.62
LULC	Moderately suitable	2	Cropland	2770.74	62.17
	Least suitable	3	Trees & Flooded vegetation	216.28	4.85
	Not suitable	4	Waterbody & Built Area	506.33	11.36

**Figure 3:**
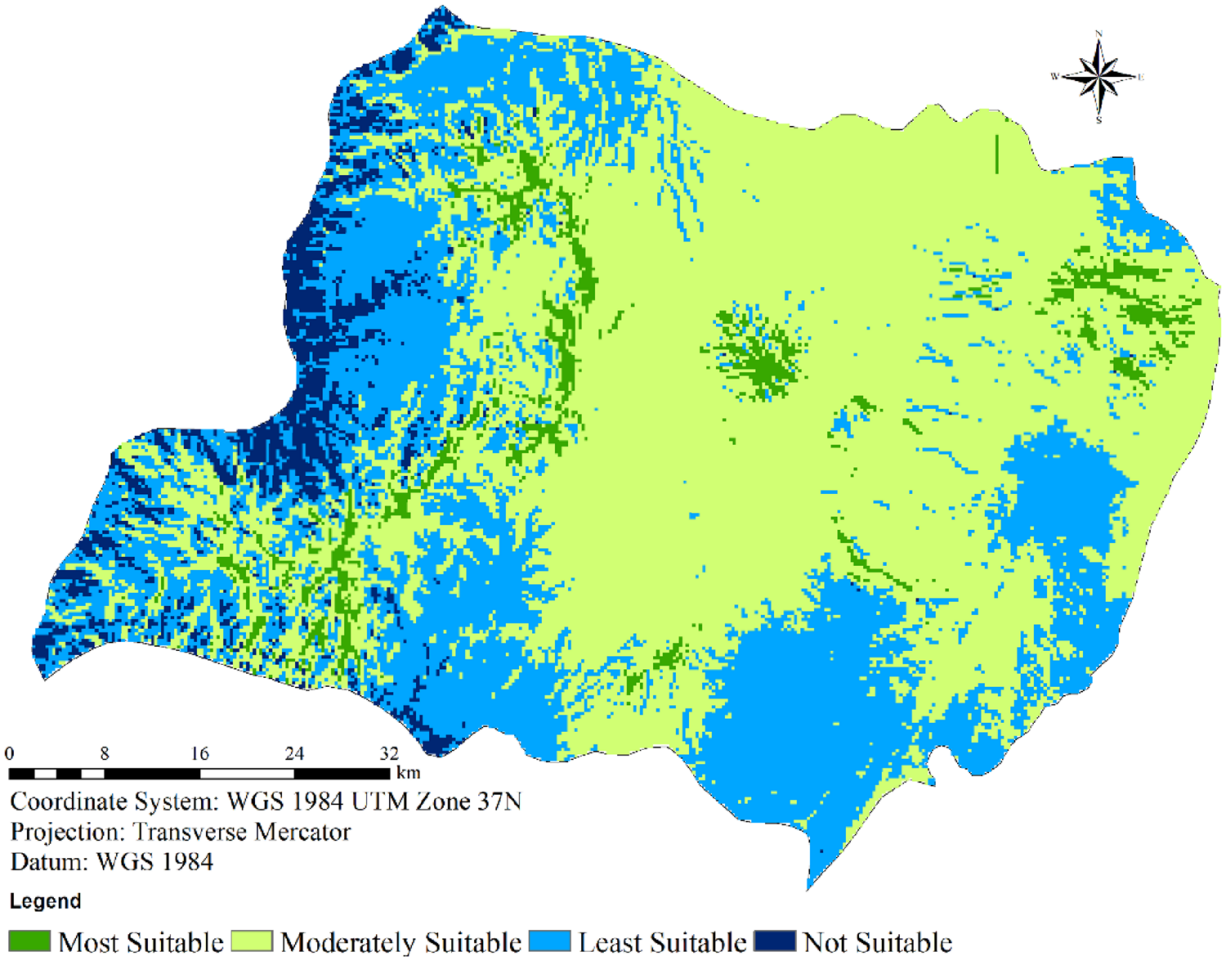
Wind speed suitability for wind farms.

Based on the wind speed map, it was determined that approximately 65.16% of the entire area presents a favorable environment for the establishment of wind farms (as depicted in Fig. 3). Among the area’s composition, about 4.69% features high wind speeds exceeding 3.5 m/s, while 60.47% experiences wind speeds in the moderate range of 1.75–3.5 m/s. In contrast, around 29.05% of the area is categorized as having the least suitability, and an additional 5.79% is considered unsuitable for the installation of wind power plants (Table 2).

Therefore, locations with stronger winds are better suited than those with weaker winds; Fig. 3 illustrates the geographic ranges of wind speed suitability. The central, northeastern, northwestern, southeastern, southwestern, and southern tip regions of the study area are better suited for wind farms than other regions.

#### Distance to settlements

**Figure 4:**
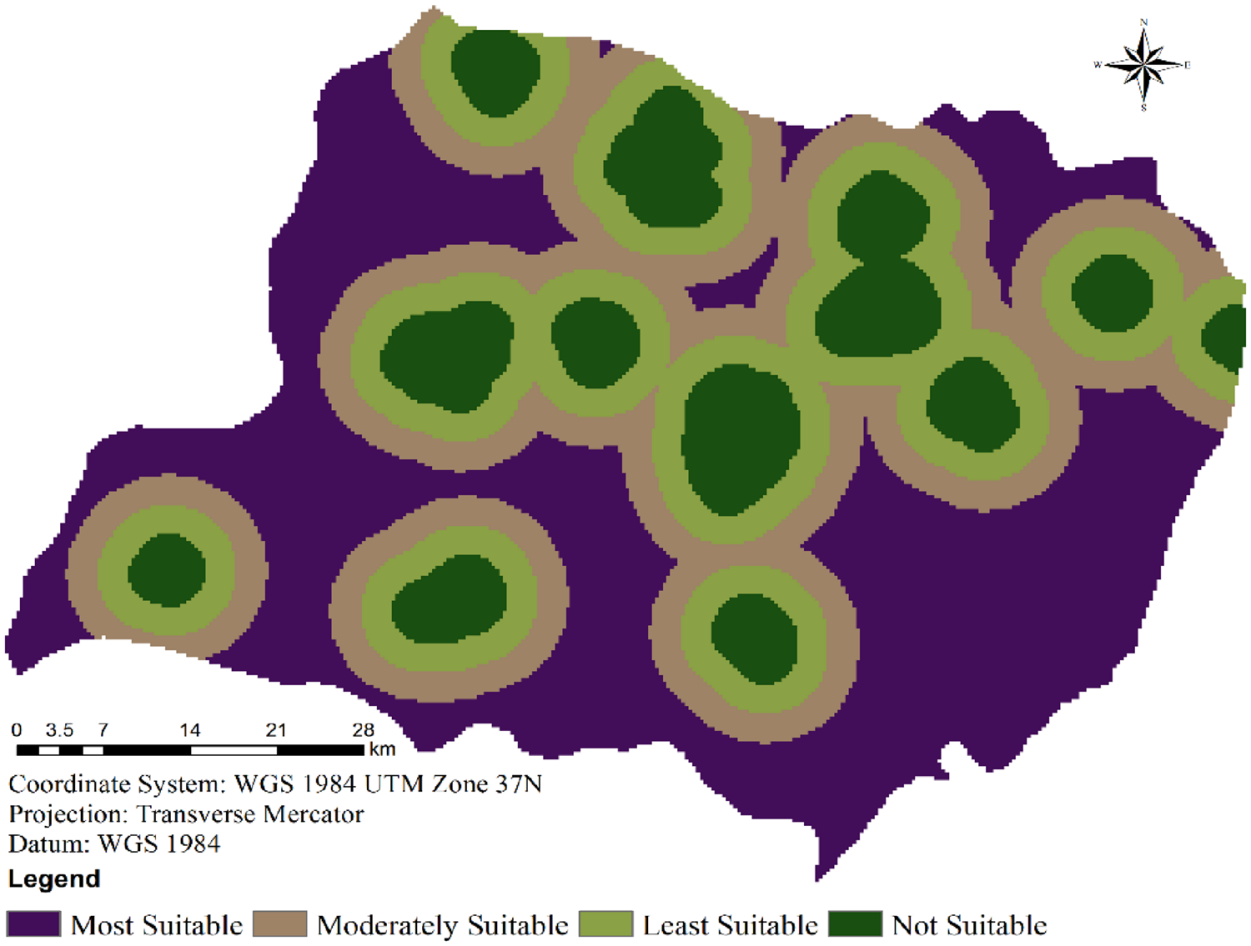
Suitability of wind farms in terms of distance from settlements.

The proximity of wind farms to residential areas can give rise to noteworthy challenges. Noise, shadow flicker, visual concerns, and aesthetic impacts stand out as primary issues in the development of wind farms. In order to mitigate noise and visual disturbances, it is necessary to establish wind farms at specific distances from urban communities. In this evaluation, the distances from settlements were redefined into four categories, drawing on data sourced from the central statistics agency (CSA), zonal capitals, and woreda capitals (as illustrated in Fig. 4 and detailed in Table 2)^12,28^. Consequently, distances of less than 2.5 km were classified as unsuitable, while distances exceeding 10 km were regarded as highly suitable (Table 2). Additionally, the ranges of 2.5–5 km, 5–7.5 km, and 7.5–10 km were categorized as less suitable, moderately suitable, and suitable, respectively (Table 2).

Overall, the wind farm site under consideration proves to be well-suited for wind energy development, largely due to the fact that over half of the area is situated more than 10 km away from settlements (Fig. 4). This outcome could potentially stem from the limited presence of highly urbanized communities within the study area.

#### Distance to power lines (PL)

The proximity of potential wind farm sites to transmission lines stands as another pivotal consideration, profoundly influencing both the establishment and functioning of such a facility. To comprehensively address this aspect, all existing transmission lines were meticulously factored into this evaluation. As a result, areas in close proximity to transmission lines are identified as highly favorable, whereas those situated at greater distances are categorized as unsuitable^25,31^.

**Figure 5:**
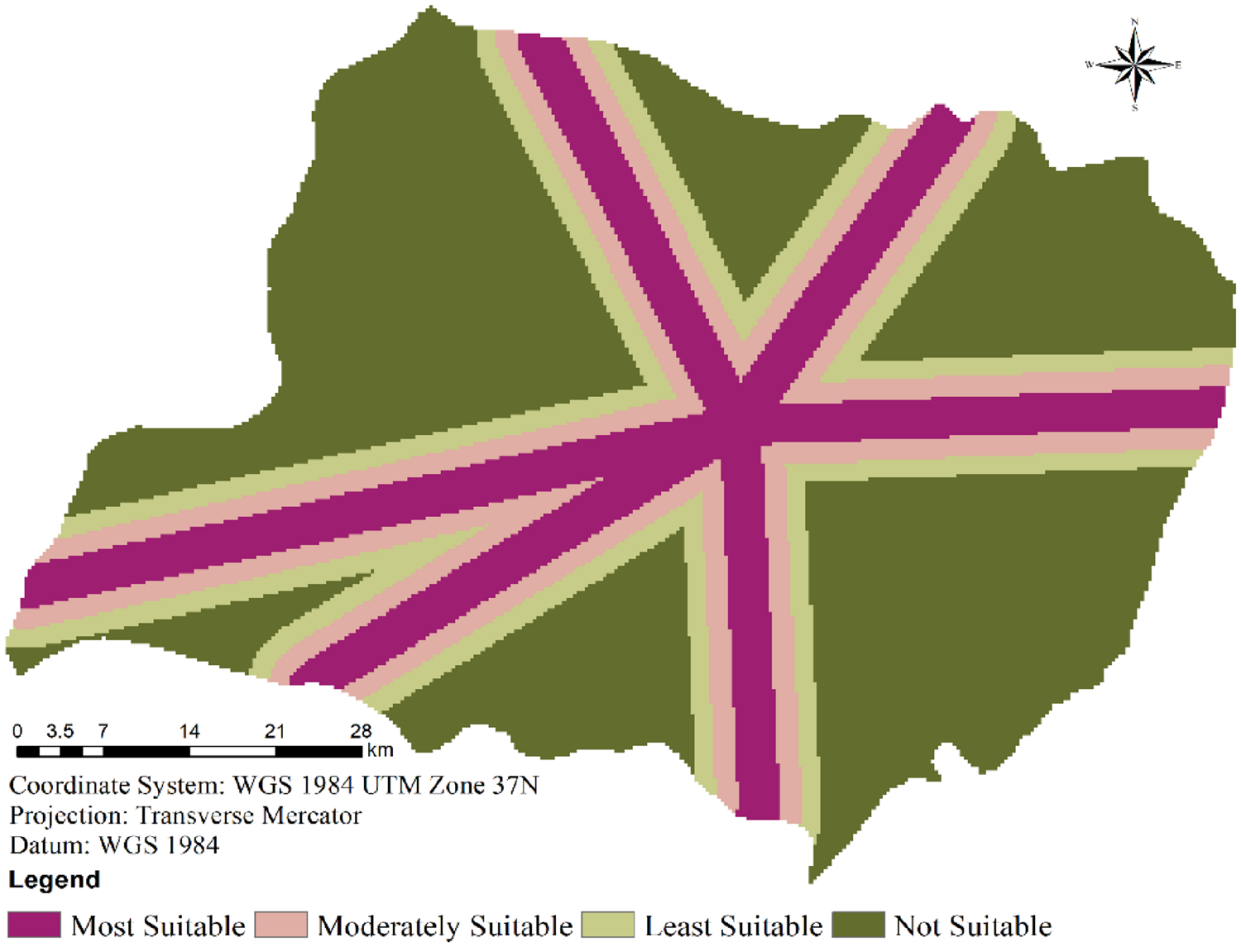
Suitability of wind farms in terms of distance from PLs.

As indicated in Table 2, within this study, the term “highly suitable” refers to locations less than 1.67 km away from Power Lines (PLs), while distances exceeding 5 km are considered inappropriate. Similarly, the categorization of PL suitability includes “moderately suitable,” “less suitable,” and “unsuitable” for distances spanning 1.67–3.34 km, 3.34–5 km, and beyond 5 km, respectively. According to the insights provided by Table 2 and visually depicted in Fig. 5, approximately 33.74% of the overall area is classified as suitable in the context of transmission line proximity, leaving the remaining portion designated as unsuitable.

#### Distance to rivers

The ecological functions attributed to water bodies render them incompatible with the establishment of wind farm sites^32^. Consequently, a considerable separation between wind turbines and water bodies is imperative to safeguard both the ecological integrity of the water bodies and the operational stability of the turbines. To address this concern, previous studies have established guidelines. For instance^41^, stipulated a minimum distance of 250 m from rivers and surface water, while^42^ implemented a 400 m zoning setback from water bodies.

**Figure 6:**
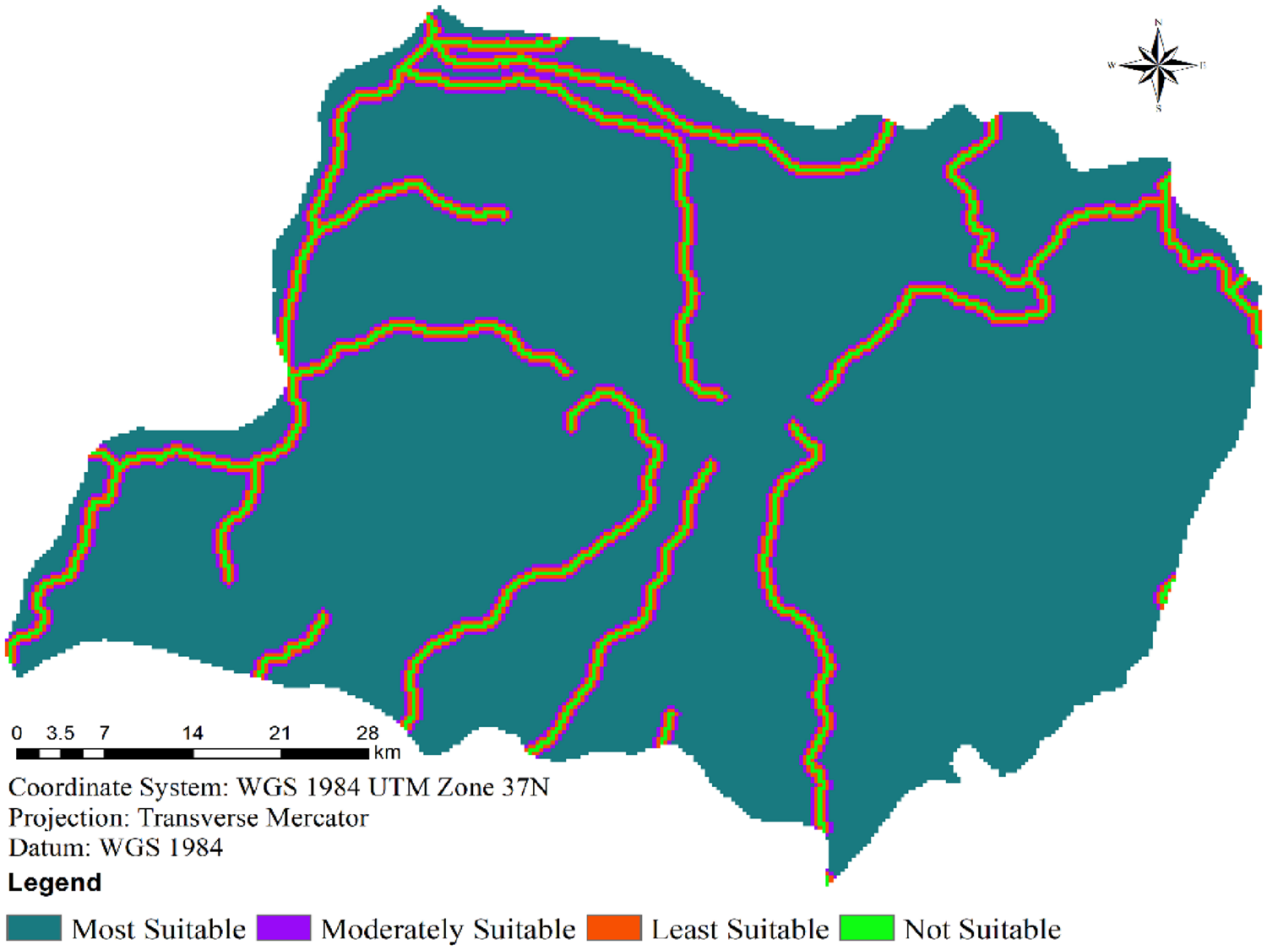
Suitability of wind farms in terms of distance from rivers.

In the context of this study, a protective buffer of 1 km was designated around lakes, and a 200 m buffer was established around rivers. This strategy led to the identification of areas exceeding 600 m from water bodies as suitable for wind farm construction. This distinction is visually represented in Fig. 6, illustrating the distance from water bodies. The map vividly portrays that upwards of 90% of the land area lies within a range of less than 600 m from a water body, consequently rendering it highly favorable for wind farm installation. In contrast, a limited 3.80% of the land area is considered unsuitable for wind farm development.

#### Distance to transportation network

When selecting suitable sites for wind farms, one must factor in the accessibility of the area to major transportation networks^34^. Optimal utilization of pre-existing roads contributed to cost reductions in wind farm construction. The presence of established roads facilitates site access, whereas the creation of new roads substantially escalates production costs^35^. To ensure the efficient transportation of turbines and equipment, it is essential to ascertain the existence of accessible roadways or the feasibility of establishing them at a reasonable expense.

**Figure 7:**
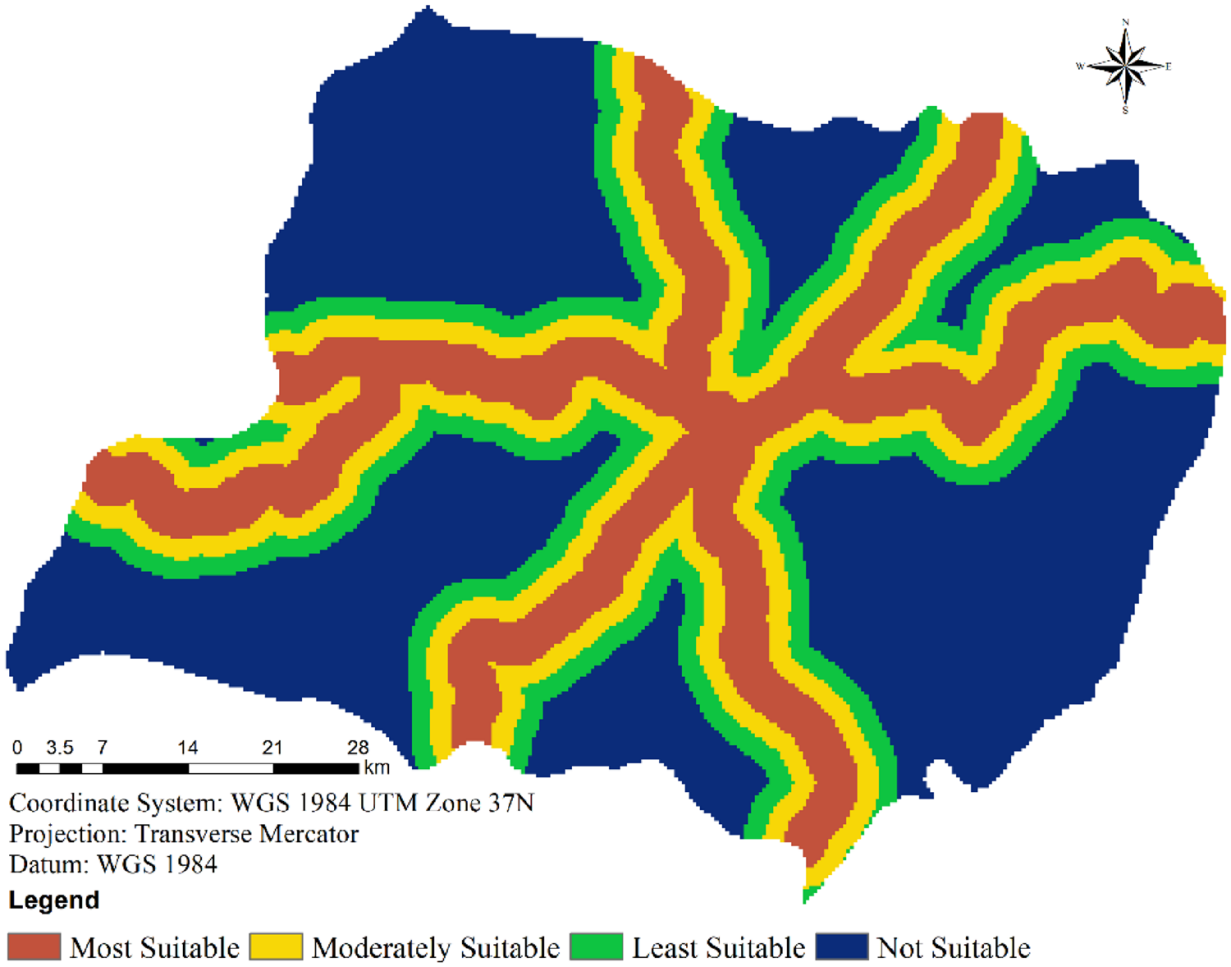
Suitability of wind farms in terms of distance from transportation network.

Within this study, the distance from roads was categorized into four distinct classes, utilizing the Euclidean distance technique within spatial analyst methods (as presented in Table 2 and depicted in Fig. 7). In line with Table 2, locations situated within a 1.67 km radius of a street were regarded as highly suitable, while those positioned farther than 5 km were deemed unsuitable. This distinction originates from the understanding that constructing new roads and transporting wind turbines becomes increasingly costly with greater distances from existing road networks^34,35^.

Figure 7 graphically illustrates the road connectivity layout within this context. Notably, approximately 20.71% of the total land area lies within a distance of less than 1.67 km from a road, making it an ideal candidate for wind farm placement. Furthermore, an additional 16.99% of the total area falls within the range of 1.67–3.34 km from roads, signifying moderate suitability for wind energy development. Conversely, a significant 47.82% of the area is deemed unsuitable for wind farm construction, due to inadequate road access.

#### Slope

The topographical incline of the terrain significantly impacts the ease of wind turbine installation and maintenance, thereby constituting a crucial factor in site selection. The choice of an appropriate location is closely tied to the slope’s effect, given that erecting and sustaining wind turbines on steep terrain presents greater challenges and escalates construction expenses within the study area^28^. Additionally, the transportation of cranes and turbines becomes considerably intricate in areas characterized by steep slopes.

**Figure 8:**
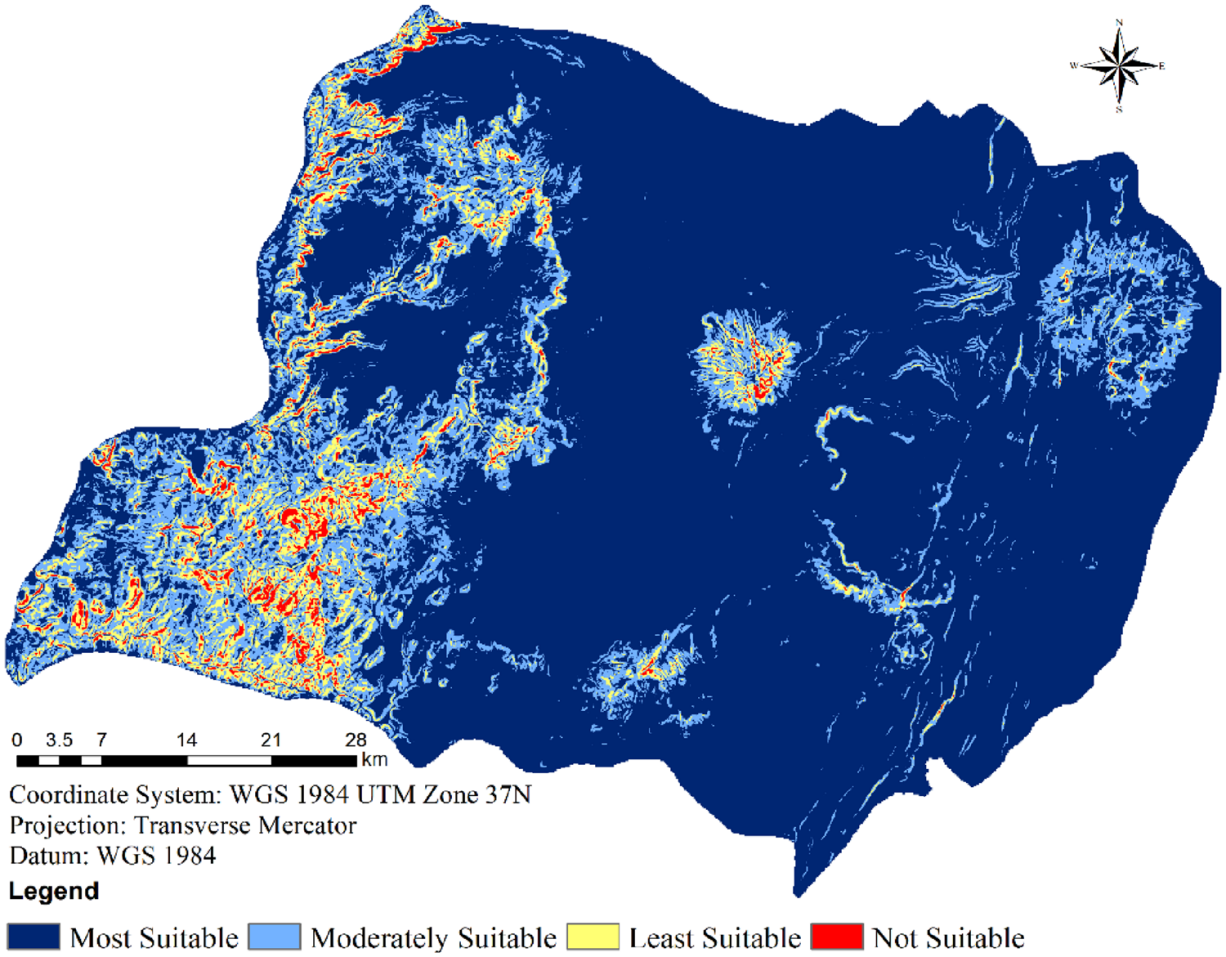
Suitability of wind farms in terms of Slope.

Within the context of this study, a steep slope is deemed unsuitable, while a gentle slope is considered suitable^34,35^. Employing the digital elevation model of the SRTM with a 30-m resolution, a slope map was generated using GIS technology. Subsequently, the slope raster was categorized into four distinctive classes based on slope degree. In this instance, it was determined that slopes below 10 degrees are highly suitable, while slopes exceeding 10 degrees are deemed unsuitable (Table 2)^34,35^. The slope map presented in Fig. 8 visually illustrates how the predominant portion of the study area, encompassing 75.70% of the total land area, is highly suitable for wind farm development due to the gentle nature of its slopes.

#### Land use/Land cover (LULC)

**Figure 9:**
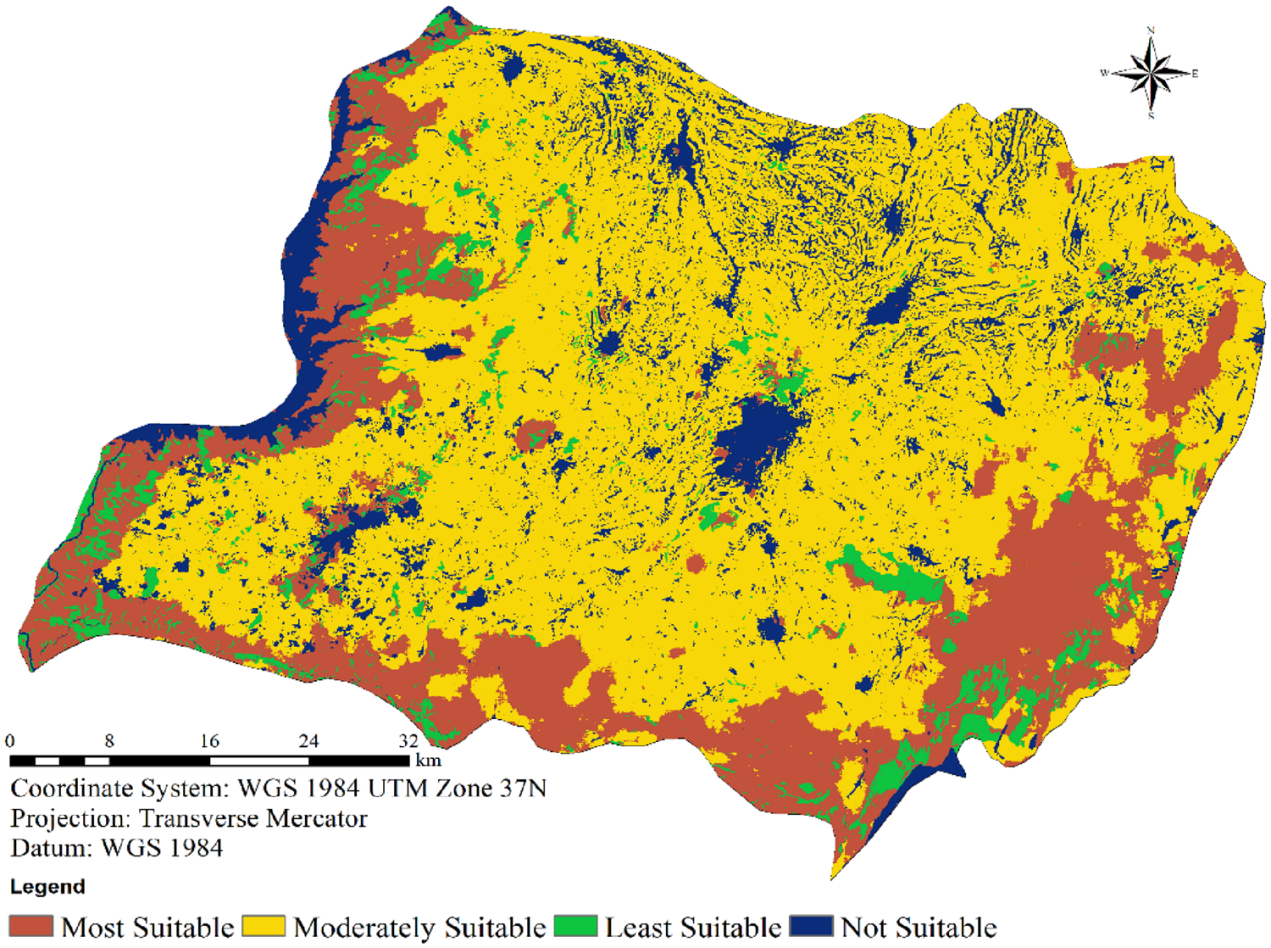
Suitability of wind farms in terms of LULC.

The predominant land cover type plays a crucial role in determining the feasibility of establishing wind turbines in a particular location. Beyond technical considerations, the acceptability of wind farm installations within a social context is influenced by varying perceptions of different land cover types. Consequently, the ongoing land use pattern in areas rich in wind resources holds pivotal significance in the strategic selection of wind farm sites. Notably, certain regions that exhibit ample wind potential may still be unsuitable for wind farm construction due to factors such as environmental preservation or public interest. For instance, locations encompassing wetlands, historical sites, aviation zones, and military areas often restrict wind farm establishment, despite their favorable wind conditions^34,35^. In light of this, it can be broadly posited that land types like agricultural expanses, grasslands, barren terrains, and shrub-covered areas are particularly well-suited for wind farm setups, while forested regions are considered less suitable^34,35^. To maintain congruence with appropriateness and ethical considerations, our study deliberately excluded wetlands, water sources, and settlement areas from consideration for wind farm development^23^. Consequently, our assessment categorized croplands and bare land as highly and moderately suitable land classes, respectively, whereas wetland/settlement/waterbody areas and forests were labeled as less suitable and unsuitable, respectively (as indicated in Table 2). Additional analysis revealed that croplands accounted for 62.17% of the total area, while bare land comprised 21.62%. Conversely, forested and wetland/settlement/waterbody regions constituted 4.85% and 11.36% of the overall area, respectively, as depicted in Table 2 and Fig. 9. This comprehensive evaluation of land cover proportions further enriches our understanding of the study's findings, casting light on the spatial distribution and significance of various land cover categories within the study area.

### Wind farm suitable areas

In this section, we outline the method of determining factor weights and suitability analysis for wind farm site selection. Using AHP and spatial analysis, we assess variable significance and their combined impact on suitability. We interpret AHP results, discuss suitability categories, and present a comprehensive suitability map, offering insights into optimal wind farm locations in Wolaita.

#### Interpretation of AHP results

The determination of cumulative weights for wind energy suitability variables was executed utilizing the ArcMap mapping software. This involved the integration of AHP tools to establish the weightage of each factor. A pair-wise comparison matrix was employed to express relative preferences among these factors. Through this matrix, weights were attributed to each factor, reflecting their prioritization or the assessment of one factor’s significance about another. However, it’s worth noting that in certain instances, complete consistency might not prevail across all criteria pairs during comparison. To gauge the reliability of the criteria comparison, the consistency ratio (CR) was employed. A CR value less than 0.1 signifies a reasonable level of consistency in the pair-wise comparisons^25^. If the CR considerably surpasses 0.1, the process may be deemed ineffective or necessitate a revision, as the considerations might be too closely linked to randomness.

To ensure a pragmatic level of accuracy, a few assumptions can be set up to subsequently evaluate the remaining data. The AHP weight outcomes for each element are summarized in Table 3. Notably, the variable of wind speed criteria garnered the highest priority weight at 43.29%, aligning with studies conducted in Adama. On the other hand, proximity to settlements and slope inclination were both found to have a minor impact on wind farm establishment, each holding a weight of 5% (Table 3). By the findings of this study, the calculated consistency ratio (CR) stands at 0.06, a value deemed acceptable as it falls below the threshold of 0.10.

**Table 3:** Weighing scores for each criterion.

	AHP (%)	Weighted overlay (%)
Reclassified wind speed	43.29	43
Reclassified existing electric grid lines	19.75	20
Reclassified existing roads	13.57	14
Reclassified distance to settlement	4.76	5
Reclassified slope	5.32	5
Reclassified river	6.87	7
Reclassified LULC	6.44	6

#### Wind farm suitability categories

**Figure 10:**
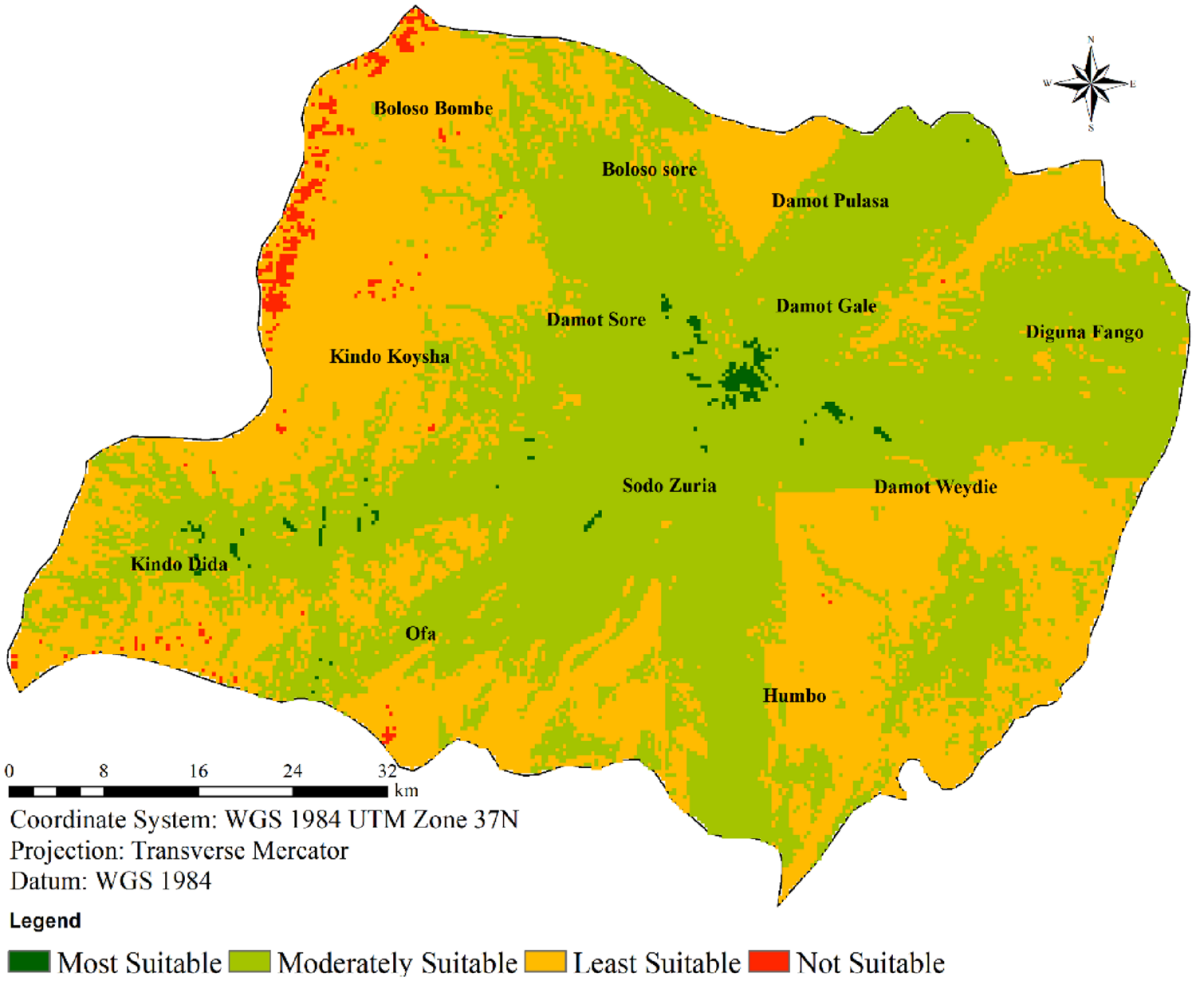
Final wind farm suitability map for Wolaita.

Utilizing a weighted overlay approach within ArcMap, the comprehensive wind farm suitability map for the study region was generated, as outlined in Table 4. The final map is divided into four distinct classes, as depicted in Fig. 10. Analyzing the data presented in Table 4, it becomes evident that a mere 0.628% of the entire area, equivalent to 28.00 km^2^, is categorized as highly suitable for the establishment of wind farms. In contrast, a substantial area of 2433.96 km^2^, constituting 54.61% of the land, falls into the category of moderate suitability. Conversely, a modest 0.85% (37.85 km^2^) of the total expanse is deemed unsuitable for hosting wind turbines, leaving a sizable 43.91% (1956.94 km^2^) classified as less suitable.

**Table 4:** Final suitability score and area coverage for Wolaita.

Suitability score	Classes	Area (km^2^)	Area coverage (%)
Most suitable	1	28.00	0.628192
Moderately suitable	2	2433.96	54.61289
Least suitable	3	1956.94	43.90959
Not suitable	4	37.85	0.849328
		4456.74	100.00

Both Table 4 and Fig. 10 provide insight into the distribution across categories, revealing that a significant portion of Wolaita (comprising 54.61% of the total area) is moderately suitable. The most favorable locations are concentrated in the central, northwestern, and southern sectors of the study area. Given these outcomes, it can be concluded that there exists ample room for the deployment of wind farms within the study region, especially if sites exhibiting high or moderate suitability are harnessed.

A comparison analysis of similar studies carried different locations using GIS-AHP methods was provided to support the conclusions of this study and to give stakeholders a global perspective (Table 5).

**Table 5:** Review of selected similar studies.

Location and year	Aim	Factors	Constraints	Weighting /ranking	Refs.
Egypt, 2017	Assess offshore wind potential and identify suitable sites	Wind speed, bathymetry, distance to shore/grid, seabed	Shipping, ports, military zones, protected areas, cables, oil/gas	AHP using pairwise	^43^
Greece, 2017	Identify suitable sites for hybrid offshore wind and wave energy systems	Wind speed, wave power, water depth, distance to shore, grid connection, population served	Military areas, hydrocarbon areas, existing renewable sites, protected areas	AHP	^44^
Songkhla Province, Thailand, 2018	Identify ideal sites for utility-scale wind and solar farms	Climate, topography, land use, buffers, proximity, and farm area	Minimum wind speed, slope, distance buffers for airports, settlements, etc	AHP	^45^
Indonesia, 2019	identify suitable locations for wind farm construction	Wind speed, infrastructure, land cost, population, electricity demand, natural disasters	Minimum wind speed, maximum natural disaster risk	hierarchical fuzzy DEA	^46^
Indiana, USA, 2016	develop a GIS-based framework for identifying suitable sites for utility-scale wind farms	Forests, streams, development, slopes, roads, rail, power lines, airports, habitats, historic sites	Minimum distances from forests, streams, settlements; maximum slope; wind speed threshold	fuzzy + AHP	^47^
Serbia, 2023	develop a GIS and PROMETHEE based method for selecting optimal wind farm locations	Distance to protected areas, settlements, infrastructure, land use, ownership, community acceptance etc	sensitive areas, minimum distances from settlements	fuzzy + AHP	^48^
Sudan, 2022	Identify optimal sites for wind farm installation	Wind speed, slope, distance from transmission lines, distance from cities, distance from airports, elevation, distance from roads/railways, lightning strike flash rate	low wind speed, high slope, high elevation, proximity to transmission lines, proximity to cities, proximity to airports, proximity to roads, high lightning strike rate	Fuzzy AHP	^49^
Wolaita, Southern Ethiopia, 2022	Assess wind farm site suitability	Wind speed, distance to settlements, distance to power lines, distance to rivers, distance to roads, slope, land use/land cover	Minimum wind speed, maximum slope, minimum distances from settlements, rivers, roads etc	AHP	This study

## Conclusion

Ethiopia's commitment to a climate-resilient green economy, aiming to enhance power supply and reduce greenhouse gas emissions by 2030, underscores the significance of this study. This initiative aligns with climate resilience and green economy principles, essential for energy source diversification and mitigating the impacts of erratic rainfall and severe droughts on hydropower. This study demonstrates the effectiveness of using GIS and AHP to identify suitable locations for wind energy projects in the Wolaita area, Ethiopia. GIS integrated with the AHP proves to be a resilient and effective methodology for selecting suitable locations for wind energy projects, considering diverse socio-economic and environmental factors. The findings from this study can be summarized as follows:

- The study highlights significant potential in various sections of the study area, including the central, northeastern, northwestern, southeastern, southwestern, and southern regions.
- A precise proportion of 0.628% of the entire area, equivalent to 28.00 km^2^, is highly conducive for wind farm construction. Approximately 54.61% (1329.11 km^2^) of the total land area, amounting to 2433.96 km^2^, is moderately suitable.
- In contrast, 0.85% (37.85 km^2^) of the total area is deemed inappropriate for wind turbine installation. This assessment indicates that about 43.91% (1060.00 km^2^) of the territory exhibits a lower level of appropriateness for wind turbine deployment.
- The study’s results affirm that the study region possesses significant potential for wind farm installations by effectively utilizing locations that are highly and moderately appropriate.

This study establishes a foundation for future investigation, specifically focusing on the unique geological characteristics of the region, intending to enhance our comprehension and optimize the implementation of wind energy projects. As we progress into the future, our discoveries have the potential to inspire and mobilize people from all sectors, leading to increased participation in Ethiopia's efforts towards sustainable energy. The combined endeavors will guide the country toward a future characterized by reduced carbon emissions, a more environmentally sustainable path, and a stronger energy infrastructure. This will serve as a significant model for policymakers, the global scientific community, and industrial players.

## Data Availability

The datasets generated and/or analyzed during the current study are available from the corresponding author on reasonable request.
